# Τhe Greek Variant in APP Gene: The Phenotypic Spectrum of APP Mutations

**DOI:** 10.3390/ijms222212355

**Published:** 2021-11-16

**Authors:** Stefania Kalampokini, Despoina Georgouli, Eleni Patrikiou, Antonios Provatas, Varvara Valotassiou, Panagiotis Georgoulias, Cleanthe Spanaki, Georgios M. Hadjigeorgiou, Georgia Xiromerisiou

**Affiliations:** 1 Medical School, University of Cyprus, Nicosia, Cyprus and Department of Neurology, General Hospital of Nicosia, Nicosia 2029, Cyprus; hadjigeorgiou.georgios@ucy.ac.cy; 2 Department of Neurology, University Hospital of Larissa, 41334 Larissa, Greece; despgeorgo@gmail.com (D.G.); antoinepr2004@hotmail.com (A.P.); georgiaxiromerisiou@gmail.com (G.X.); 3 Department of Rheumatology and Clinical Immunology, Faculty of Medicine, School of Health Sciences, University of Thessaly, 41223 Larisa, Greece; eleni_patrikiou@yahoo.gr; 4 Nuclear Medicine Laboratory, University Hospital of Larissa, Faculty of Medicine, School of Health Sciences, University of Thessaly, 41110 Larissa, Greece; vvalotasiou@gmail.com (V.V.); pgeorgoul@uth.gr (P.G.); 5 Department of Neurology, Medical School, University of Crete, 70013 Heraklion, Greece; kliospanaki@gmail.com

**Keywords:** amyloid precursor protein, mutation, duplication, phenotype

## Abstract

Mutations in the gene encoding amyloid precursor protein (APP) cause autosomal dominant inherited Alzheimer’s disease (AD). We present a case of a 68-year-old female who presented with epileptic seizures, neuropsychiatric symptoms and progressive memory decline and was found to carry a novel APP variant, c.2062T>G pLeu688Val. A comprehensive literature review of all reported cases of AD due to APP mutations was performed in PubMed and Web of Science databases. We reviewed 98 studies with a total of 385 cases. The mean age of disease onset was 51.3 ± 8.3 (31–80 years). Mutations were most often located in exons 17 (80.8%) and 16 (12.2%). The most common symptoms were dementia, visuospatial symptoms, aphasia, epilepsy and psychiatric symptoms. Mutations in the β-amyloid region, and specifically exon 17, were associated with high pathogenicity and a younger age of disease onset. We describe the second reported APP mutation in the Greek population. APP mutations may act variably on disease expression and their phenotype is heterogeneous.

## 1. Introduction

Alzheimer’s disease (AD) is a progressive neurodegenerative disorder characterized by the accumulation of insoluble forms of β-amyloid (Aβ) in plaques in extracellular spaces and in the walls of blood vessels as well as the aggregation of hyperphosphorylated protein tau in neurofibrillary tangles in neurons [1,2]. AD has a prevalence of 10–30% in the population over 65 years old [1]. The disease is characterized by progressive memory loss and the disturbance of other cognitive functions, namely word-finding, spatial cognition, reasoning, judgment and problem solving [2]. The disease has a long prodromal phase, which can occasionally extend over two decades and an average disease duration of 8–10 years [1].

A small proportion of patients (<1%) have autosomal dominant inherited AD [1,3]. This form is characterized by disease onset at a relatively young age (before 65 years of age) and usually positive family history for dementia [2,4]. It may manifest with atypical symptoms such as apraxia, aphasia, dyscalculia, visual symptoms or seizures [2]. It is caused by autosomal dominant penetrant mutations in the genes encoding amyloid precursor protein (APP) (OMIM 104760), presenilin 1 (PSEN1) (OMIM 104311) and 2 (PSEN2) (OMIM 600759), leading to overproduction of Aβ [1,2]. APP mutations or duplications are the second most common cause of monogenic forms of AD following PSEN1 mutations [5,6] and are responsible for approximately 15–20% of early-onset cases [4,5].

APP is a type I transmembrane protein with a large extracellular domain and a short cytoplasmic region; its coding gene is located on chromosome 21 (21q21.2-3) [7]. The APP gene contains 18 exons and encodes an alternatively spliced transcript, which in its longest isoform expresses a polypeptide of 770 amino acids [6]. Aβ is released from APP through two cleavage events, one in the extracellular area (β-secretase cleavage) and one in the transmembrane area (γ-secretase cleavage) [5,7]. If cleavage occurs at residue 712–713 the most common Aβ40 is produced, whereas if it occurs after residue 714 the longer Aβ42 is generated, which is prone to fibril formation and promotes Aβ aggregates [5]. Presenilins, the proteins encoded by PSEN1 and PSEN2 genes, are main components of the γ-secretase complex, responsible for the cleavage of APP into Aβ peptides [5,8]. PSEN mutations cause a shift of γ-secretase cleavage, increasing Aβ42 production [5,9]. APP and Aβ are the cornerstone of the ‘’amyloid cascade hypothesis’’ [10], which states that the accumulation of Aβ initiates a cascade of pathological processes such as tau hyperphosphorylation, neurofibrillary tangles formation, neuroinflammation, loss of synaptic junctions and neuronal death [4].

Here, we present a case of a female patient with disease onset at the age of 58 years who was found to carry a novel APP variant, c.2062T>G pLeu688Val. Moreover, we reviewed all existing literature reporting AD cases due to APP mutations and discuss phenotype–genotype correlations.

### 1.1. Case Report

**Figure 1:**
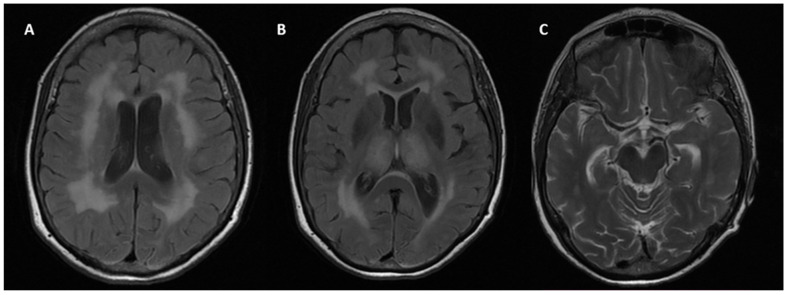
Structural MRI sequences of the patient with APP mutation presenting with Alzheimer’s disease. (**A**,**B**) Axial Flair with severe leukoencephalopathy and symmetrically increased signal intensity in the thalamus (**C**) Axial T2-weighted MRI image showing hippocampal atrophy.

**Figure 2:**
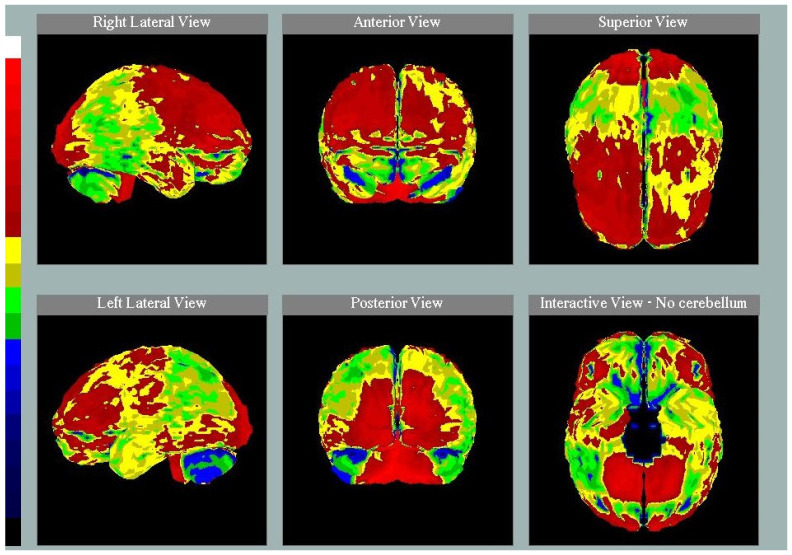
Single-photon emission computed tomography (SPECT) of the patient with the use of Stereotactic Surface Projection (SSP) method and cerebellum as reference standard, showing bilateral hypoperfusion of the parietal and posterior temporal lobes. The color scale on the left side of the image represents perfusion in the brain cortex compared with perfusion of cerebellum in descending order from the top (red color, corresponding to normal perfusion) to the bottom (blue color, corresponding to severely reduced perfusion).

We describe the case of a 68-year-old woman, who was admitted to the Neurological Department of the University Hospital of Larissa in Greece with severe dementia for further examination. She developed epileptic seizures as the first symptom at the age of 58. The patient has also developed depression and anxiety in the past 5 years according to her husband. The neurological examination revealed brisk tendon reflexes, bilateral Babinski sign and palmomental reflex. The patient’s Mini-mental state examination (MMSE) score was 11/30. Brain magnetic resonance imaging (MRI) revealed severe leukoencephalopathy and global brain atrophy especially in the temporal region and hippocampus (Figure 1). Brain perfusion single-photon emission computerized tomography (SPECT) was performed. The evaluation of perfusion in the whole brain cortex was made using the Stereotactic Surface Projection (SSP) method and the cerebellum as reference, which is considered to be the least affected area in degenerative dementias [11,12]. The SPECT study revealed hypoperfusion in the posterior temporal and parietal lobes, bilaterally (Figure 2). EEG showed epileptic discharges mainly located on the temporal lobes. Routine cerebrospinal fluid (CSF) testing was normal. She reported a family history of young onset dementia and gait disorder in her father as well as dementia in her older brother (75 years old).

Based on the MRI findings, a full screening including complete blood count, erythrocyte sedimentation rate, glucose level, renal and liver function tests, serologic tests for syphilis, vitamin B12, folate levels, thyroid function tests, HIV serology, lupus anticoagulant, antiphospholipid antibodies, antinuclear and antineutrophil cytoplasmic antibodies, were performed. Cardiac echocardiography, holter monitoring and carotid ultrasound showed normal findings.

During her second evaluation, 7 years ago, a next generation sequencing (NGS) panel for leukodystrophy and leukoencephalopathy was performed without any pathogenic variants. The patient was put on levetiracetam 1000 mg twice daily and was evaluated again a year later. In the meantime, she developed difficulties with calculation, showed disorientation in new surroundings and progressive memory decline. Her relatives reported that she had become impatient and irritated. Detailed neuropsychiatric examination revealed severe memory impairment, concentration difficulties, disorientation, visuospatial deficits and depression. Based on the cognitive deterioration, we performed a CSF analysis examining the validated AD CSF biomarkers Aβ1-40, Aβ1-42, total tau (T-tau), and phosphorylated tau (P-tau181) as well as 14-3-3 and total prion protein (t-PrP), that revealed an AD profile with decreased Aβ42/Aβ40 ratio and increased T-tau (T-Tau 634 ng/L (normal range <400), P-Tau 75 ng/L (<80), Aβ42 546 ng/L (>330), Aβ40 12,456 ng/L (>330), Aβ42/Aβ40 ratio 0.043 (>0.7)). Clinical examination, assisted by SPECT scan, as well as CSF biomarkers classified this patient as mixed dementia, that is, a combination of AD and vascular dementia.

### 1.2. Genetic Analysis

In view of the above findings, the positive family history and the early onset dementia, a genetic screening of the patient and her family (daughter, siblings) was performed after written informed consent. Our study was approved by the Ethics Committee of the University Hospital of Larissa. Genomic DNA of all available family members was extracted from peripheral white blood cells according to standard protocol. We performed whole exome sequencing (WES); sequencing raw data was conducted on Torrent Suite 5.10 software using default parameters. The resulting variants (vcf file) were imported for filtering, prioritization and evaluation into the ClinGenics Exome Management Application-EM^®^ pipeline software (v.1.0.0.1) (ClinGenics Ltd., London, UK). Selected clinically significant variants were confirmed by standard DNA Sanger sequencing. Primer sequences and polymerase chain reaction conditions are available upon request.

## 2. Methods

### 2.1. Review of the Literature

**Figure 3:**
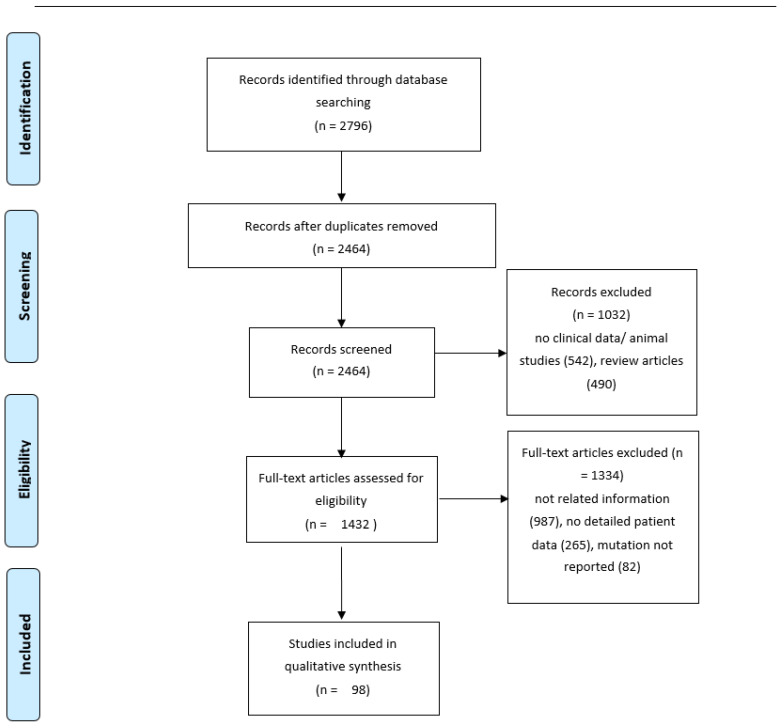
Flow-Chart of included studies in the review.

A comprehensive literature review of all reported cases of AD due to APP mutations was performed. We searched PubMed and Web of Science databases in English using the following search terms: “Alzheimer’s disease”, “dementia”, “APP or Amyloid precursor protein mutation”, “APP or Amyloid precursor protein duplication” “APP or Amyloid precursor protein gene” in order to identify all published studies in humans before May 2021. Additional articles were identified by hand-searching of the references of included articles. The flow chart of the studies included in this review can be seen in Figure 3. We included 98 studies with a total of 385 cases. Patients’ characteristics such as gender, ethnicity, age at presentation, age of onset, symptoms, MMSE score, family history of dementia, imaging findings as well as mutation type were retrieved from the included studies.

### 2.2. Statistical Analysis

The analysis was carried out with SPSS version 25.0. We used descriptive statistics to calculate the demographic, clinical and imaging characteristics of patients. Data were checked for deviation from normal distribution with the Shapiro-Wilk normality test. Categorical data were analyzed with χ^2^ test and continuous data with one-way ANOVA. 

The evaluation of Pathogenicity of Mutations was carried out using Computational Prediction. The Combined Annotation Dependent Depletion (CADD) algorithm was used for scoring the deleteriousness of single nucleotide variants as well as insertion/deletions on the function of APP [13,14]. Prediction is based on empirical rules applied to the sequence, phylogenetic, and structural information characterizing the amino-acid substitution (https://cadd.gs.washington.edu/, accessed on 5 May 2021). 

APP gene protein regions were classified as region 1 or first domains (exons 1–6), region 2 or Kunitz-type protease inhibitor (KPI) region (exons 7–8), region 3 (exons 9- part of 16), region 4 or Aβ region (part of exon 16 and exon 17) and region 5 or cytoplasmic region (part of exon 17-exon 18). V717I or G mutation was calculated separately due to the high percentage of patients carrying this mutation (region 6). For all the analyses, a 5% significance level was set.

## 3. Results

### 3.1. Genetic Analysis

**Figure 4:**
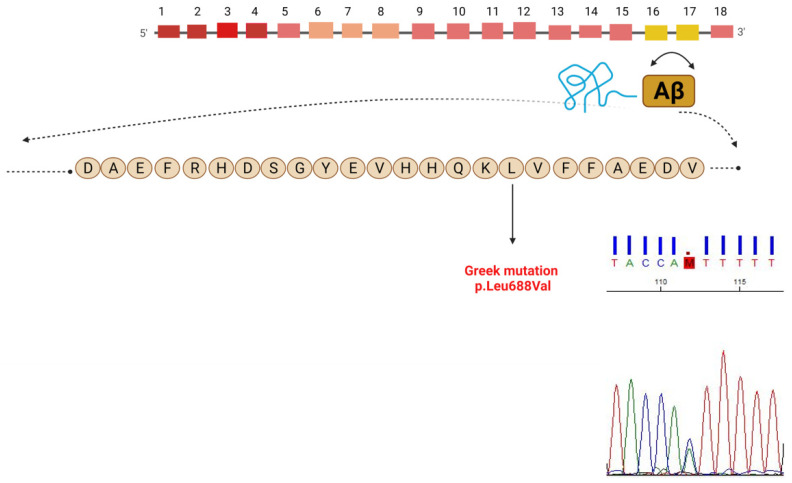
The APP gene consisting of 18 exons is located on chromosome 21 (21q21.2-3) and is alternatively spliced into several products, named according to their length in amino acids. The region encoding the amyloid sequence comprises part of exons 16 and 17. The Greek mutation and the related chromatogram are shown with the black arrow.

We detected an APP variant, c.2062T>G p.Leu688Val which has been reported in international databases previously as a disease-causing mutation. Cosegregation analysis by Sanger sequencing confirmed the presence of this variant in the patient. The patient’s father unfortunately has died and was not tested. The patient’s older brother was also found to carry the mutation, while her younger sister (64 years old) was asymptomatic and did not have the mutation. Her daughter (44 years) was also found to carry the mutation but did not exhibit any symptoms at the time of testing. According to the ACMG-AMP 2015 guidelines, the pathogenicity potential of the variant is “likely pathogenic” based on the following criteria: (a) The same missense change at an amino acid reside has been described before in another Greek family with dementia phenotype (PS1) (b) The absence of the variant from controls in Exome Sequencing Project, 1000 Genomes Project or Exome Aggregation Consortium (PM2) (c) In silico bioinformatics tools (Homologene, GEPR, Varsome) predicted that the variant causes a deleterious effect on the gene (PP3) as it occurs in a highly conserved area across multiple species (http://www.ncbi.nlm.nih.gov/homologene, accessed on 5 May 2021) (d) the patient’s phenotype and family history is highly specific for a disease with a single genetic etiology (PP4). This novel Greek mutation on APP gene and the related chromatogram are shown in Figure 4.

### 3.2. Demographics and Clinical Phenotype

We identified 385 cases of AD due to APP mutations. From those, APP duplications were found in 48 cases (12.5%). Almost half of the mutations (170, 52.1%) were found in Asian patients, 126 (38.7%) in Caucasian, 20 (6.1%) in Latin/Hispanic and 10 (3.1%) in African patients. The distribution of mutations according to gender was equal, half (49.7%) were male and half (50.3%) female. The mean age of disease onset was 51.3 ± 8.3 years (range 31–80 years). One hundred and four studies reported a family history of dementia. The mean MMSE score was 17 ± 8.1 at the time of examination. The Combined Annotation Dependent Depletion (CADD) score was 27.8 ± 3.2 (moderate pathogenicity). CADD score correlated weakly with age of onset (r = −0.168, *p* = 0.005), i.e.; increased CADD score was associated with younger age of onset.

**Figure 5:**
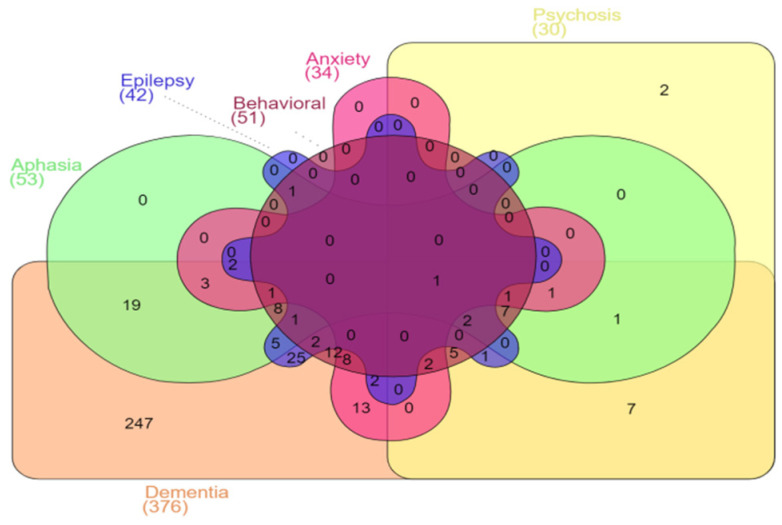
Common symptoms of APP mutation carriers from the described cases in the literature presented in a Venn diagram. Note: Each symptom is represented by a specific color and shape, e.g.; dementia is represented by a pink rectangle, aphasia by a green infinity shape, behavioral symptoms by a brown circle, epilepsy by a blue cogwheel, anxiety by a red cross and psychosis by a yellow rectangle. The areas where two colors and shapes are crossed represent the cases with both symptoms.

The most common symptom was dementia, reported in 376 cases (97.7%). Other symptoms were visuospatial reported in 112 cases (29.1%), aphasia in 53 (13.8%) and epilepsy in 42 (10.9%). Other reported symptoms were extrapyramidal (24 cases, 6.2%), pyramidal (21 cases, 5.5%), myoclonus (18 cases, 4.7%), apraxia (13 cases, 3.4%), ataxia (12 cases, 3.1%) and dyscalculia (7 cases, 1.8%). With regard to psychiatric symptoms, depression was reported in 52 cases (13.5%), anxiety in 34 (8.8%) and psychotic symptoms in 30 (7.8%). Behavioral symptoms were reported in 51 cases (13.2%). A diagram of common symptoms is presented in Figure 5. 

### 3.3. Neuroimaging

One hundred twenty-one studies reported brain atrophy and 32 hippocampal atrophy. Leukoencephalopathy on MRI was reported in 47 cases. Leukoencephalopathy was not associated with any specific location of the mutation, neither protein region (χ^2^ = 6.195, *p* = 0.288) nor exon (χ^2^ = 1.278, *p* = 0.528). CSF findings with amyloid decrease were reported in 21 cases. Twenty-seven studies reported amyloid accumulation in Positron emission tomography (PET) and eight hypometabolism. SPECT showed hypoperfusion (parietal, parietooccipital, frontoparietal, parietotemporal) in 12 cases. EEG was reported abnormal (triphasic waves, slow waves, spikes or combination) in ten studies and normal in three cases. The rest of the studies did not provide relevant imaging or laboratory data.

### 3.4. Mutations

**Figure 6:**
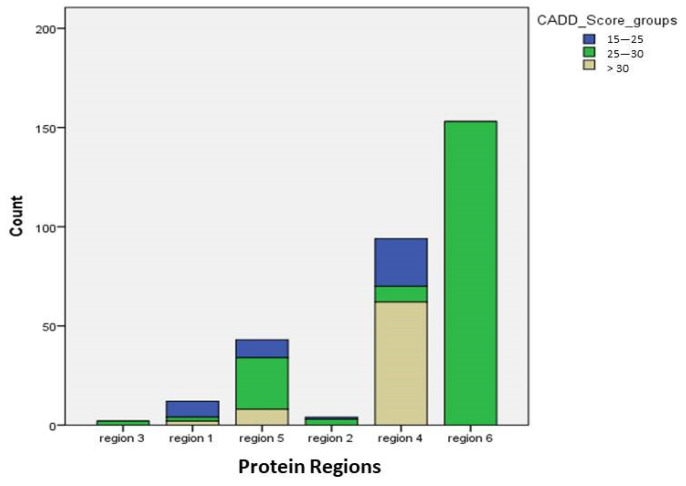
Association of protein regions of APP gene mutations with CADD (Combined Annotation Dependent Depletion) score. Note: region 1 or first domains = exons 1–6, region 2 or Kunitz-type protease inhibitor (KPI) region = exons 7–8, region 3 = exons 9–part of 16, region 4 or Aβ region = part of exon 16 and exon 17, region 5 or cytoplasmic region = part of exon 17–exon 18, region 6 = V717I mutation.

With regard to location of mutations, the mutations were located mostly in exons 17 (265 cases, 80.8%) and 16 (40 cases, 12.2%) and less frequently in exons 4, 5, 6, 7, 11, 14, 15 and introns (23 cases, cumulative percentage 6.6%). With regard to protein regions, the most common location of mutations was position 717 (153 cases, 49.5%), followed by Aβ region (95 cases, 30.7%), region 5 (part of exon 17-exon 18) (43 cases, 13.9%), region 1 (exons 1–6) (12, 3.9%), KPI or region 2 (4 cases, 1.3%) and region 3 (exons 9- part of 16) (2 cases, 0.6%). We found an association between protein regions and CADD score (χ^2^ = 259.51, df = 10, *p* < 0.001). Mutations in Aβ region were associated with higher CADD score, while mutations in 717 position or region 5 with moderate CADD score. Mutations in region 1 were associated with low CADD score (Figure 6). We also found an association between CADD score and exon mutation position (χ^2^ = 140.75, df = 4, *p* < 0.001*). More specifically, mutations in exon 17 were associated with a moderate to high CADD score, while mutations in exon 16 or any other exon with low or moderate CADD score. Patients with mutations in region 1 (first domains of the protein) had a later age of onset (56.9 ± 8.6) while patients with mutations in region 5 (cytoplasmic region) the youngest age of onset (49.3 ± 13.7). No association was found between exon mutation position and age of onset. MMSE score did not correlate with neither protein region nor exon position of the mutation. Moreover, MMSE score did not correlate with CADD score. 

With regard to ethnicity, the majority of Caucasian (58, 61.7%) and Asian patients (160, 97%) had a mutation in exon 17. All Latin/Hispanic (18, 100%) and Africans (10, 100%) had a mutation in exon 17. Latin and African had CADD score of moderate severity (25–30), Asian and Caucasian had mostly moderate (65.4% and 64.5% respectively) and severe CADD score (28.9% and 12.9% respectively). There was no association between ethnicity and age of onset. 

## 4. Discussion

### 4.1. Clinical Spectrum of APP Mutations: Age of Onset and Symptoms

The patient that we described presented with epileptic seizures and extensive leukoencephalopathy. Our patient’s mutation is located on the protease cleavage site of APP, on the Aβ domain, where the majority of pathogenic mutations have been described so far, close to the Iowa and Dutch mutation (Figure 4). Approximately 400 cases of APP mutations have been described so far, the majority of which are located on the Aβ domain or referring to the V717I, F or G mutation. Another case with the same mutation was recently described in a Greek patient as well [15]. This case had hereditary cerebral amyloid angiopathy with occipital calcifications, progressive cognitive decline and motor symptoms. As several mutations tend to be exclusive in certain populations we named this mutation the “Greek variant”.

We found that the mean age of disease onset of APP mutation carriers was 51.3 (±8.3) years, similar to previous published review studies and meta-analyses [3,16]. Disease onset of APP mutation carriers commonly ranges between 45 and 60 years [5,17]. There was also a reported case with disease onset even in the eighth decade [18]. In fact, families carrying the same APP mutation [19,20,21] have a significantly different age at onset, suggesting that other genetic or environmental modifiers of the disease may exist [16]. Moreover, there are significant differences between mutation types, resulting in some cases in onset in the third or fourth decade of life [16]. The youngest individual with APP mutation causing AD [22] was a patient with a positive family history of early-onset AD, disease onset at the age of 31 and death at age 36. He harbored the APP I716F mutation [23]. With regard to this mutation, in vitro studies showed a marked increase in the Aβ42/40 ratio, suggesting reduced APP proteolysis by γ-secretase [24]. These findings strengthen the inverse association between Aβ42/40 ratio and age of onset [24]. 

The most common symptoms of APP mutation carriers in our analysis were cognitive symptoms and/or dementia (almost 98% of cases). In fact, the majority of monogenic AD cases have an amnestic presentation [5]. Early neuropsychological findings are deficits in verbal memory with relatively preserved naming and object perception, executive dysfunction and disorientation [25,26]. Visuospatial symptoms were also very common, occurring in almost one third of patients. Other cortical symptoms such as aphasia and apraxia were less common; aphasia occurred in less than 20% of patients. Indeed, atypical language presentation is rather rare in APP cases [5]. Seizures, on the other hand, were present in approximately 10% of patients and may represent the first presentation in monogenic AD cases [5], as in our patient’s case. Indeed, amyloid β-peptides can induce neuronal hyperexcitability and trigger epileptic seizures [27]. Furthermore, we found that a small proportion of patients (about 10%) presented with pyramidal (spasticity, hemiparesis, paraparesis) or extrapyramidal symptoms (mostly rigidity). Notably, extrapyramidal symptoms are very rare in APP mutation carriers and tend to appear after several years of disease [5,17]. Other movement disorders, such as ataxia, myoclonus or rest tremor, were also rare. With regard to psychiatric symptoms, depression and anxiety were encountered in less than one quarter of the patients. Other psychotic or behavioral symptoms, such as delusions, hallucinations, or aggression—which are common in sporadic cases—can also be found in monogenic APP cases [3,5]; however, as shown in our analysis they are rather rare. Concerning APP duplications carriers, apart from dementia, they may present with seizures and other focal cortical symptoms such as aphasia, apraxia and dyscalculia, extrapyramidal, pyramidal or behavioral symptoms [28,29,30,31]. 

Our patient had severe leukoencephalopathy on MRI. Notably, certain APP mutations [19,32,33,34,35,36,37,38] and duplications [28,29] have been associated with variable white matter abnormalities up to leukoencephalopathy. The APP mutations associated with leukoencephalopathy were within the Aβ sequence [34,35] such as the Iowa mutation [32], near β-sekretase [33,37] or γ-sekretase [19,36,38] cleavage site. The age of onset was early in those cases (39–57 years) and they presented with both typical (dementia) and atypical i.e.aphasia, apraxia, seizures, psychiatric) symptoms. 

### 4.2. Mutations in Amyloid Precursor Protein (APP) Gene: Location and Pathogenicity

Most APP mutations are missense or nonsense mutations within or flanking the Aβ sequence and near the cleavage sites of secretases [2]. More specifically, we confirmed that most APP mutations (93%) are located on exons 16 and 17, which constitute the transmembrane Aβ region and encode the Aβ sequence. This was observed independently of ethnicity, although data from African and Latin/Hispanic populations are limited. In most AD families due to APP mutation, the inheritance pattern is autosomal dominant, while homozygous carriers do not seem to be more severely affected [39]. However, recessive APP mutations have also been reported [40,41]. The amino acid position can, in fact, predict pathogenicity [23]. Indeed, we showed that mutations in exon 17 are associated with moderate to severe pathogenicity (CADD score), while mutations in exon 16 or other exons with mild to moderate pathogenicity. Additionally, mutations in the Aβ protein region were associated with severe pathogenicity, unlike mutations in the cytoplasmic or 717 positions, which were associated with moderate pathogenicity. Patients with mutations in the cytoplasmic region had the youngest age of onset in our analysis (before the age of 50). These findings are reasonable, as these mutations affect the area encoding the Aβ sequence. Furthermore, duplications of variable size have been identified [2]. However, APP duplications are far less frequently reported than missense mutations [2]. APP duplications display reduced penetrance and higher variability in age of onset, compared to missense mutations, which show a near-complete disease penetrance [42]. The phenotype of APP duplications is not associated with the size of duplication [43]. 

Mutations in exons 16 and 17 alter the processing of the protein and cause the accumulation of Aβ42 fragments by decreasing Aβ40 peptide levels or increasing Aβ42 production [44,45]. Our patient was found to harbor a missense mutation located at position 688 of APP, between the β- and γ-secretase cleavage site. Most pathogenic mutations of APP occur near the β-secretase cleavage site (amino acids 670aa–682aa), near the γ-secretase cleavage site (amino acids 713aa–724aa) or in the Aβ sequence (amino acids 692aa–705aa) [43]. Mutations within the Aβ domain such as the Dutch [46,47], Flemish [48] and Iowa mutation [32] have variable effects on APP processing such as impaired α-secretase cleavage and increased aggregation of Aβ into fibrils [7,43]. Mutations in the C-terminal (e.g., in exon 17) influence the activity of γ- and e-secretases and result in a selective increase in the production of longer Aβ peptides, especially Aβ42, which are more hydrophobic and prone to the formation of fibrils [10,43]; here belongs the I716F mutation (Iberian) associated with the youngest age-at-onset [22,24]. Moreover, a mutation in APP that decreases the production of Aβ (A673T) [49] has been shown to have protective effects on late-onset AD. This substitution results in an approximately 40% reduction in the formation of amyloidogenic peptides in vitro. The protective effect of A673T substitution against AD supports the hypothesis that reducing β-cleavage of APP may be an effective primary preventive strategy. Therefore, the Aβ42/40 ratio could be a useful indicator of the aggressiveness of the mutation [23].

### 4.3. Phenotypic Variability of APP Mutations

AD is a disease with phenotypic variability, especially in cases with early disease onset. APP gene mutations may act variably on disease expression, ranging from high penetrance (causal allele) and early age of onset to low penetrance (risk allele) and late-onset, depending on the effect of the mutant allele on protein function [50]. Different mutations at the same APP locus can segregate with different transmission patterns, that is, dominant, semi-dominant and recessive [37,40,41,51]. It is to be discovered whether this is associated with specific characteristics of the single mutations or genetic modifiers in those families [51]. Apart from pathogenic mutations, genetic variations, such as single nucleotide variants (SNVs) in the promoter region of APP, have been associated with increased susceptibility for AD [2]. In addition, epigenetic or other genetic factors may play a role in clinical variability of individuals carrying the same mutation [4]. Epigenetic dysregulation (DNA methylation, chromatin remodeling, non-coding RNAs expression) can affect gene expression in AD such as alteration in methylation in the promoter region of APP [52].

## 5. Conclusions

In summary, the phenotype of APP mutation carriers is heterogeneous. The age of disease onset ranges from the 40 s to 70 s. The main symptoms in patients harboring APP mutations are cognitive, while focal cortical, extrapyramidal symptoms, seizures, behavioral and psychiatric symptoms can also occur. Data from families that segregate a monogenic form of AD and patients with a known causal mutation provide the opportunity to identify mutation-specific effects and link genotypic changes with clinical and pathophysiological manifestations of the disease. In the future, different genetic causes of AD should be targeted with specific interventions. Asymptomatic carriers of APP mutations can also serve as candidates for disease-modifying treatment or prevention trials. Moreover, another direction for future research should be the identification of genetic and environmental modifiers of disease onset and progression [16]. Studying the mechanisms underlying these mutations can provide more insight into the pathways leading to AD pathology, in order to plan appropriate intervention strategies for the disease. 

## Data Availability

Data and genetic analysis (primer sequences and polymerase chain reaction conditions) are available upon request.
